# Development of a measure of dietary quality for the UK Biobank

**DOI:** 10.1093/pubmed/fdad103

**Published:** 2023-06-29

**Authors:** Chloe Montague, Stefania D’Angelo, Nicholas Harvey, Christina Vogel, Janis Baird

**Affiliations:** Health Education England Wessex, Winchester SO21 2RU, UK; MRC Lifecourse Epidemiology Centre, Southampton General Hospital, University of Southampton, Southampton SO16 6YD, UK; MRC Lifecourse Epidemiology Centre, Southampton General Hospital, University of Southampton, Southampton SO16 6YD, UK; MRC Lifecourse Epidemiology Centre, Southampton General Hospital, University of Southampton, Southampton SO16 6YD, UK; NIHR Southampton Biomedical Research Centre, University of Southampton and University Hospital Southampton NHS Foundation Trust, Southampton SO16 6YD, UK; MRC Lifecourse Epidemiology Centre, Southampton General Hospital, University of Southampton, Southampton SO16 6YD, UK; NIHR Applied Research Collaboration Wessex, Southampton Science Park, Innovation Centre, 2 Venture Road, Chilworth, Southampton SO16 7NP, UK; MRC Lifecourse Epidemiology Centre, Southampton General Hospital, University of Southampton, Southampton SO16 6YD, UK; NIHR Southampton Biomedical Research Centre, University of Southampton and University Hospital Southampton NHS Foundation Trust, Southampton SO16 6YD, UK; NIHR Applied Research Collaboration Wessex, Southampton Science Park, Innovation Centre, 2 Venture Road, Chilworth, Southampton SO16 7NP, UK

**Keywords:** food and nutrition, circulatory disease, dietary pattern

## Abstract

**Background:**

Previous studies of the UK Biobank have examined intake of single food items and their association with health outcomes. Our aim was to develop a dietary quality score and examine the relationship between this score and markers of cardiometabolic health.

**Methods:**

Principal component analysis was performed on dietary data from UK Biobank participants. Linear regression was used to analyse the relationship between diet and cardiometabolic health.

**Results:**

The first component explained 14% of the variation in the dietary data. It was characterised by high consumption of meat and low fibre carbohydrates, and a low intake of fruit and vegetables. A higher score, indicative of healthier diet, was associated with lower systolic and diastolic blood pressure (β −0.81, 95% CI −1.0, −0.62; β − .61, 95% CI −0.72, −0.5) and a healthier lipid profile (lower levels of cholesterol β −0.05, 95% CI −0.06, −0.04, triglycerides β −0.05, 95% CI −0.06, −0.03, and higher HDL cholesterol β 0.01, 95% CI 0, 0.01).

**Conclusions:**

The dietary quality score was a good approximation of overall dietary quality. An unhealthy diet was associated with markers of poorer cardiometabolic health.

## Introduction

Poor diet, defined as one that is low in fruits, vegetables, whole grains, nuts and seeds, and high in sodium, sugar and trans-saturated fat, is the single biggest contributor to poor health globally.^1^^,^^2^ Poor diet is a risk factor for many non-communicable diseases, in particular, cardiovascular disease, type 2 diabetes and many cancers, and often clusters with other behaviours such as smoking and physical inactivity.^2^^,^^3^

When examining population diet and health outcomes, there is increasing emphasis on the assessment of diet as a whole, or dietary patterns, rather than single nutrients or food groups. Measurement of dietary patterns allows for the fact that foods are eaten in combination and the balance of nutrients in a person’s diet is an important determinant of disease.^4^ Techniques for measuring dietary intake include food diaries, dietary recalls and food frequency questionnaires (FFQ).^5–7^ Weighted food diaries are the gold-standard of dietary assessment but they are time consuming and costly.^6^^,^^7^ Dietary recall methods, such as FFQs, offer a less resource intensive alternative approach that is designed to capture both habitual diet and irregular consumption.^8^ FFQs comprise a list of food and/or drink items and the participant is asked to state how often they consume each item over a period of time.^5^^,^^8^ They can be used quickly and easily in studies with large numbers of participants.^8^

One of the benefits of analysing dietary data using dietary patterns is that it can provide a better understanding of overall dietary quality and, in turn, reveal stronger associations with health outcomes.^9–11^ Two approaches have been used for dietary patterns analysis. The first of these (an a priori approach) compares dietary intake to an existing framework, to assess adherence to dietary recommendations or a specific type of diet. The second (a posteriori) approach is data-driven, using dietary data to identify common patterns of consumption.^12^ Principal component analysis (PCA) is one such approach and can identify key foods, both healthy and unhealthy, that contribute most strongly to a dietary pattern.

Several studies have investigated the feasibility and usefulness of data-driven approaches to measure dietary patterns in middle- to older-aged adults.^13–15^ These studies, for the most part, demonstrated a positive association between a diet high in fruits and vegetables and/or a negative association between a diet high in meat and processed foods with body mass index (BMI) and/or waist circumference.^13–15^

The UK Biobank is a large prospective cohort study, incorporating a wide range of health, lifestyle and genetic data from over half a million participants with links to their ongoing health records.^16^ It provides a unique opportunity to examine the determinants of health and investigate a range of diseases and health outcomes in a group of middle- to older-aged adults. The quantity of dietary data available within the UK Biobank is extensive. Previous studies have often employed a single food item or food group as an approximation of dietary quality.^17–21^ Five studies have examined red meat and processed meat as a determinant of disease.^17–21^ Collectively, results showed that higher levels of red and processed meat intake were associated with a higher risk of ischaemic heart disease, ischaemic stroke, pneumonia, diverticular disease, colon polyps, diabetes and colorectal cancer.^17–19^ Applying a measure of dietary quality in such investigations could further strengthen associations between diet and health outcomes by better representing this multidimensional behaviour.

Harnessing FFQ data collected in the UK Biobank to describe dietary patterns may offer a more nuanced approach to analysing diet-disease relationships that complements approaches using single food groups. The first aim of the present study was to apply PCA to UK Biobank FFQ data to identify a dietary pattern that could describe overall dietary quality within the study. The secondary aim was to evaluate this new dietary quality score by assessing its relationship with objective markers of cardiometabolic health and other modifiable determinants of disease. The link between dietary quality and cardiovascular disease is well established.^22^ It was therefore hypothesised that an unhealthy diet score would be associated both with markers of cardiometabolic health and other determinants of disease, such as smoking and physical inactivity.

## Methods

### Participants

This study used data from the UK Biobank.^23^ Participants were invited by letter to take part in the UK Biobank between 2006 and 2010 if they were registered with the NHS, aged between 40 and 69 years, and living within a reasonable travel distance of one of the assessment centres.^23^ More than 500 000 men and women have been recruited from England, Scotland and Wales.^23^ The Greater London Foodscapes sample was comprised of 52 345 UK Biobank participants who were recruited to one of the three London centres.^24^ Of these 52 345 participants, 33 149 provided complete dietary data and were therefore included in this analysis.

### Procedure

At the baseline assessment, UK Biobank participants completed a touchscreen questionnaire, had their physical measurements taken, and provided blood, urine and saliva samples.^23^

### Demographic data

Demographic information including sex, age, ethnicity, level of education and Index of Multiple Deprivation (IMD) was obtained at the baseline assessment.^24^

### Health behaviour data

Physical activity and smoking status data were collected at the baseline assessment. Participants were asked about the type and duration of physical activity and their current smoking status.^25^

### Dietary data

During the baseline assessment, participants completed an FFQ detailing their usual frequency of intake of 17 food and drink items.^26^ They reported how many times per day they consumed fruits (fresh and dried), vegetables (cooked, salad and raw), water, tea and coffee.^26^ Cereal and bread items were reported by type and frequency of consumption per week.^26^ For all other items (oily and non-oily fish, processed meat, poultry, beef, lamb/mutton, pork, cheese), participants reported their usual intake over the past year.^26^ Two further food items were derived from the data for the cereal and bread categories. Participants were allocated as either high fibre cereal consumers (e.g. bran, biscuit cereal, muesli or oat) or low fibre cereal consumers (e.g. cornflakes or Frosties). Similarly, brown/wholemeal bread and white bread were derived from the ‘bread type’ variable. This produced 19 food and drink items in total.

### Outcome measures

The outcomes of interest were physical and biochemical factors associated with a greater risk of cardiometabolic disease. Blood pressure and BMI were selected as physical measures of risk for cardiometabolic ill-health. Key biochemical markers relating to cardiometabolic health were selected to represent the biochemical outcomes of interest for this analysis, including: cholesterol, high-density lipoprotein (HDL) cholesterol, apolipoprotein A, apolipoprotein B, lipoprotein A, triglycerides, glycated haemoglobin (HbA1c), C-reactive Protein (CRP) and vitamin D.

### Statistical analysis

The baseline characteristics of the 33 149 participants included in this study were compared to the full Greater London foodscapes sample (*n* = 52 345) and to the participants with incomplete dietary data (*n* = 19 196) using either a *t*-test, a Mann–Whitney test or a chi-square test, depending on the nature of the variable.

To address the first research aim, PCA was performed on the weekly frequencies of consumption of the 19 food and drink items. PCA is a data reduction method that produces new variables that are linear combinations of the original dietary variables and maximises the explained variance within the data.^27^ The PCA analysis was based on the correlation matrix in order to adjust for unequal variances of the original variables. The first component of the PCA, the main axis of variation in the dietary data, was used to create a summary dietary quality score for each participant. These scores were created by multiplying the PCA coefficients for each of the food and drink items by each participant’s frequency of consumption, and then summing this value across all 19 items. The score was then transformed using Fisher-Yates normal scores to create a normal distribution across the sample, with a mean of 0 and a standard deviation of 1. The median of the score was used to categorise diet as healthier or less healthy. For ease of interpretation, the coefficients were multiplied by −1, so a positive score (above the median) indicated a healthier diet. The distributions of outcomes were checked by visual inspection and those that were not normally distributed were transformed using Fisher-Yates normal scores. Further details on how the PCA was performed can be found in Table 1 in the Supplementary Material.

Linear regression was used to examine the association between dietary quality and markers of cardiometabolic health with estimates presented in their unadjusted form and following adjustment for pre-defined confounding factors. Potential confounding variables were pre-defined using a Directed Acyclic Graph (DAG), with the software DAGitty.^28^ According to the DAG (see Figure 1, Supplementary Material), associations needed to be adjusted for sex, age, ethnicity, level of education, smoking and IMD.

Finally, clustering of unhealthy behaviours was analysed by cross-tabulations and chi-squared tests and included measures of smoking status, physical activity and diet quality. All analyses were performed using the statistical software Stata v15.1.

## Results

The baseline demographic characteristics of the Greater London foodscapes sample, the sample included in the analyses and participants with incomplete dietary data (who were excluded from the analysis) are shown in Table 1. For our sample, under half (45%) were male and the average age was 56.2 years (SD = 8.2). The vast majority (83.8%) of the sample was of white ethnicity and almost half had a college or university degree (49.5%). Over half of participants (51.9%) were physically active and 9.7% were current smokers. The average BMI was 26.8 kg/m^2^ (SD = 4.7). Compared with participants with incomplete dietary data, the sample included in the analyses featured a higher proportion of participants who were: male; older; of White ethnicity; physically active; non-smokers; educated to a tertiary level; living in less deprived areas; and who had a lower BMI. These differences were all statistically significant.

**Table 1:** Baseline characteristics of the sample populations

Demographic and lifestyle factors					
		Greater London foodscapes sample	Sample included in the analysis	Participants with incomplete dietary data	*P*-value^a^
n		*n* = 52 345	*n* = 33 149	*n* = 19 196	
Sex, men (%)		44.0%	45.0%	42.3%	<0.001
Age, mean (SD)		56.0 (8.2)	56.2 (8.2)	55.6 (8.3)	<0.001
White ethnicity, (%)		80.0%	83.8%	73.2%	<0.001
More than 1 day/week vigorous activity, (%)		50.4%	51.9%	47.6%	<0.001
Current smokers, (%)		11.8%	9.7%	15.6%	<0.001
Level of education, (%)					
College or University degree		47.8%	49.5%	44.7%	<0.001
Other prof qual/HND/A levels		26.8%	26.8%	26.7%	
GCSE or less		25.4%	23.7%	28.6%	
Index Multiple Deprivation, median (IQR)		18.1 (10.0, 27.4)	16.5 (9.4, 26.3)	20.5 (12.0, 29.6)	<0.001
**Markers of cardiometabolic health**					
*n*		52 345	33 149	19 196	
Systolic blood pressure, mmHg mean (SD)		134.4 (18.1)	134.2 (17.9)	134.7 (18.5)	0.002
Diastolic blood pressure, mmHg mean (SD)		81.0 (10.1)	80.8 (10.0)	81.5 (10.3)	<0.001
Cholesterol, mmol/L mean (SD)		5.6 (1.1)	5.6 (1.1)	5.6 (1.1)	0.14
HDL cholesterol, mmol/L mean (SD)		1.5 (0.4)	1.5 (0.4)	1.5 (0.4)	0.16
Apolipoprotein A, g/L mean (SD)		1.6 (0.3)	1.6 (0.3)	1.6 (0.3)	0.31
Apolipoprotein B, g/L mean (SD)		1.0 (0.2)	1.0 (0.2)	1.0 (0.2)	0.83
Lipoprotein A, nmol/L median (IQR)		23.8 (10.2, 66.6)	22.9 (10.0, 64.1)	25.4 (10.4, 69.8)	<0.001
Triglycerides, mmol/L median (IQR)		1.4 (1.0, 2.0)	1.4 (1.0, 2.0)	1.3 (1.0, 2.0)	0.01
Glycated haemoglobin, mmol/mol median (IQR)		35.4 (32.8, 38.2)	35.3 (32.7, 38.1)	35.5 (33.0, 38.6)	<0.001
C-reactive protein, mg/L median (IQR)		1.2 (0.6, 2.5)	1.1 (0.6, 2.4)	1.2 (0.6, 2.7)	<0.001
Vitamin D, nmol/L median (IQR)		42.9 (29.4, 59.1)	44.4 (30.9, 60.1)	40.2 (27.1, 56.8)	<0.001
BMI, kg/m^2^ mean(SD)		26.9 (4.9)	26.8 (4.7)	27.1 (5.1)	<0.001

^a^ *P*-value comparing participants included and excluded from the analysis and estimated by *t*-test, Mann–Whitney ranksum test, or chi-squared test

The second part of Table 1 summarises participants’ cardiometabolic health markers. Systolic and diastolic blood pressures were within the normal range.^29^ Total cholesterol was slightly elevated although HDL cholesterol and triglycerides were both within the normal ranges.^30^ Levels of apolipoproteins A and B and lipoprotein A in the sample were within the normal ranges.^31^^,^^32^ HbA1c, CRP and vitamin D levels were all also within the normal ranges. Participants’ mean BMI fell within the overweight range. There were some significant differences between the sample included in the analysis and the participants with incomplete dietary data. Participants with incomplete dietary data had a significantly higher average blood pressure, lipoprotein A levels, HbA1c, CRP and BMI compared with the sample analysed. Triglycerides and vitamin D were significantly lower in participants with incomplete dietary data compared with the sample.

After PCA was performed, the first component explained 14% of the variation in the dietary data. This component was characterised by a high consumption of beef, any type of meat, white bread and low-fibre cereals, and low intakes of fresh/dried fruit, salad and cooked vegetables and was defined as an ‘unhealthy diet score’ (see Table 2, Supplementary Material).

We found an inverse association between diet quality and blood pressure: a higher dietary quality score, indicative of healthier diet, was associated with lower blood pressure (Table 2). Healthier dietary quality scores were also associated with a healthier lipid profile (lower levels of cholesterol, apolipoprotein B, triglycerides and higher HDL cholesterol), and a lower HbA1c, CRP and BMI. These associations remained statistically significant following adjustment for sex, age, ethnicity, level of education, smoking and IMD. For vitamin D, the unadjusted results identified an inverse relationship between dietary quality and vitamin D levels. After adjusting for confounders, however, this relationship showed that healthier dietary quality scores were associated with higher vitamin D levels.

**Table 2:** Association between diet quality score and markers of cardiometabolic health

	Unadjusted			Adjusted^a^		
	*n*	β (95% CI)	*p*	*n*	β (95% CI)	*p*
Systolic blood pressure, mmHg	33 083	−1.11 (−1.31, −0.92)	<0.001	31 822	−0.81(−1.00, −0.62)	<0.001
Diastolic blood pressure, mmHg	33 083	−0.78 (−0.89, −0.67)	<0.001	31 822	−0.61(−0.72, −0.50)	<0.001
Cholesterol, mmol/L	30 967	−0.02 (−0.03, −0.01)	0.001	29 802	−0.05(−0.06, −0.04)	<0.001
HDL cholesterol, mmol/L	28 507	0.04 (0.03, 0.04)	<0.001	27 445	0.01(0.00, 0.01)	<0.001
Apolipoprotein A, g/L	28 328	0.02 (0.02, 0.02)	<0.001	27 275	−0.00(−0.00, 0.00)	0.318
Apolipoprotein B, g/L	30 821	−0.01 (−0.02, −0.01)	<0.001	29 657	−0.01(−0.02,−0.01)	<0.001
Lipoprotein A, SD	25 021	0.02 (0.01, 0.03)	0.001	27 072	-0.01(−0.02, 0.00)	0.147
Triglycerides, SD	30 946	−0.09(−0.11, −0.08)	<0.001	29 783	−0.05(−0.06, −0.03)	<0.001
Glycated haemoglobin (HbA1c), SD	30 338	−0.01 (−0.02–0.00)	0.240	29 194	−0.04(−0.05, −0.03)	<0.001
C-reactive protein, SD	30 907	−0.10 (−0.12, −0.09)	<0.001	29 744	−0.12(−0.13, −0.10)	<0.001
Vitamin D, SD	29 810	−0.02 (−0.03, −0.01)	<0.001	28 691	0.02(0.00, 0.03)	0.006
BMI, kg/m^2^	32 862	−0.68(−0.73, −0.63)	<0.001	31 612	−0.67(−0.73, −0.62)	<0.001

^a^Adjusted for sex, age, ethnicity, level of education, smoking, and IMD

The proportion of current smokers was higher among participants with an unhealthy diet score (see Table 3, Supplementary Material). The proportion of physically active participants was higher among those with a healthy diet score (see Table 3, Supplementary Material). Both findings were statistically significant (*P* < .001).

## Discussion

### Main findings of this study

This study identified that a dietary quality pattern can be created from the FFQ data in the UK Biobank. The first component that resulted when PCA was performed explained 14% of the variation in diet in the sample, and showed a dietary pattern with high intakes of meat, including processed meat, white bread and low intakes of high-fibre cereals, fruit, salad and vegetables. This dietary pattern was termed ‘unhealthy’ because it was not consistent with the recommendations within the UK Eatwell Guide.^33^ The dietary coefficients derived from the PCA for each of the 19 food and drink items were consistent with advice contained within the Eatwell Guide.^33^

The dietary quality score demonstrated that a healthier diet was associated with a healthier cardiometabolic profile after adjusting for confounding variables known to influence the relationship between diet and health.^34^ Furthermore, we found that an unhealthy diet clustered with other unhealthy lifestyle factors, namely smoking and physical inactivity. Collectively, these findings suggest that our dietary quality score is a valid measure of overall dietary quality within the UK Biobank.

### What is already known on this topic

Our measure of dietary quality for the UK Biobank compares favourably with similar analyses performed amongst older adult populations in high-income countries. Thorpe et al. used PCA to identify dietary patterns amongst Australian older adults.^35^ The dietary patterns identified explained between 5.6 and 7.8% of variation in dietary intake.^35^ A poorer quality diet, typified by high consumption of red and processed meat and refined grains was associated with a higher BMI, smoking and lower levels of physical activity.^35^ Robinson et al. investigated dietary quality across the adult life course.^36^ The dietary patterns they observed explained 7% of the variation in dietary data.^36^ A high-quality diet was associated with a high intake of fruits, vegetables, high-fibre carbohydrates and yoghurt, and a low intake of processed meat, potato products and added sugar.^36^ This was similar to the dietary pattern we observed.^36^ More recently, Gao et al. have analysed dietary patterns in the UK Biobank.^37^ The researchers used the 24-hour online dietary assessment data, in contrast to the FFQ data that we have used here.^37^ They described two main dietary patterns, explaining 43 and 20% of the variance in diet.^37^ These three studies all demonstrated an association between a poorer quality diet—typified by high levels of meat, processed meat and low-fibre carbohydrates—and markers of cardiometabolic disease.^35^^,^^36^^,^^37^

### What this study adds

A strength of this study is the large sample size afforded by the UK Biobank database. The breadth of data collected in the UK Biobank permits adjustment for a wide range of confounding variables and the use of a variety of measures of cardiometabolic health. Our study used FFQ dietary data from UK Biobank because it provides a better representation of habitual diet than 24-hour food diary data, and includes frequency data for food and drink items—improving comparisons between participants. There is the potential for error to be introduced during the application of FFQs due to recall or response bias, however, this does not prevent these data being used to rank participants intake.^42^By illustration, Bradbury et al. reported high reproducibility of the FFQ administered to UK Biobank participants and found the FFQ showed greater reliability at ranking participants dietary intake than the 24-hour food diary data.^42^

### Limitations of this study

The UK Biobank sample is not representative of the wider UK population.^38^ Of the 9.2 million people who were invited to participate, 5.5% responded.^38^ Fry et al. looked at the differences between responders and non-responders.^38^ Responders were more likely to be female, were generally healthier and less likely to live in deprived areas. Comparing our study sample directly with the wider UK population, our sample was more ethnically diverse, more physically inactive, but contained fewer smokers.^39^^,^^40^ The BMI of our sample was slightly lower than the mean of participants within the Health Survey for England 2019.^41^ Overall, these comparisons present a mixed picture of how our sample compared to the wider UK population. It is likely less deprived than the whole UK population but there is not a consistent pattern in relation to demographic factors associated with poorer health outcomes. This suggests that the use of this population to create the measure of dietary quality provides reasonable general applicability to the whole UK population. Indeed, Fry et al. acknowledge that although the UK Biobank sample is not representative of the wider population, the associations observed between health behaviours and health outcomes remain useful as a reflection of patterns of disease in the adult UK population.^38^

Another limitation of this study relates to the selection of food and drink items for the FFQ. Frequently consumed foods such as biscuits, cakes, confectionary and crisps were not included nor was the list of healthier foods exhaustive (e.g. yoghurt, beans, pulses, eggs were not included). However, studies using data driven methods, such as PCA, have shown that dietary quality scores derived from short FFQs (~20 items) correlate strongly in the expected direction with dietary scores created from large FFQs (~100 items) and with objective biomarkers of nutritional status.^42–45^

## Conclusions

The dietary coefficients derived from the PCA were consistent with the Eatwell guide showing the value this dietary pattern offers to future studies using the UK Biobank. The dietary quality score developed in this study provides a useful tool for researchers wishing to incorporate diet into their investigations when using data from the UK Biobank cohort. The dietary quality score developed in this study can be easily applied to assess dietary quality as an exposure, outcome, mediating, or confounding variable in UK Biobank analyses. The ability to apply and compare this dietary quality score with other measures of morbidity and mortality within the UK Biobank dataset could be beneficial in the understanding of the relationship between diet and disease.

The findings of this large population-based study suggest that a dietary pattern with high levels of meat consumption (including processed meats, red meats and poultry) is associated with a greater risk of poor cardiometabolic health in adults. Such findings are important for government strategy for prevention of cardiometabolic disease.

## Supplementary Material

Supplementary_material_figure_1_fdad103

Supplementary_material_table_1_fdad103

Supplementary_material_table_2_fdad103

Supplementary_material_table_3_fdad103

## Notes

**Chloe Montague,** Public Health Specialty Registrar

**Stefania D’Angelo,** Statistician

**Nicholas Harvey,** Professor

**Christina Vogel,** Professor

**Janis Baird,** Professor
