# Correction to: Development of a measure of dietary quality for the UK Biobank

**DOI:** 10.1093/pubmed/fdad280

**Published:** 2023-12-18

This is a correction to: Chloe Montague, Stefania D'Angelo, Nicholas Harvey, Christina Vogel, Janis Baird, Development of a measure of dietary quality for the UK Biobank, *Journal of Public Health*, Volume 45, Issue 4, December 2023, Pages e755–e762, https://doi.org/10.1093/pubmed/fdad103

In the originally published version of this manuscript, the following acknowledgement was omitted:

The linked Hospital Episode Statistics data are Copyright © 2023, NHS England and were used in this publication with the permission of the NHS England and UK Biobank.

This error has been corrected online.

