# Supplementary figures and images for: Development of a measure of dietary quality for the UK Biobank
Source: J Public Health (Oxf). 2023 Jun 29;45(4):e755–62. doi: 10.1093/pubmed/fdad103 (PMC10687865; doi:10.1093/pubmed/fdad103)

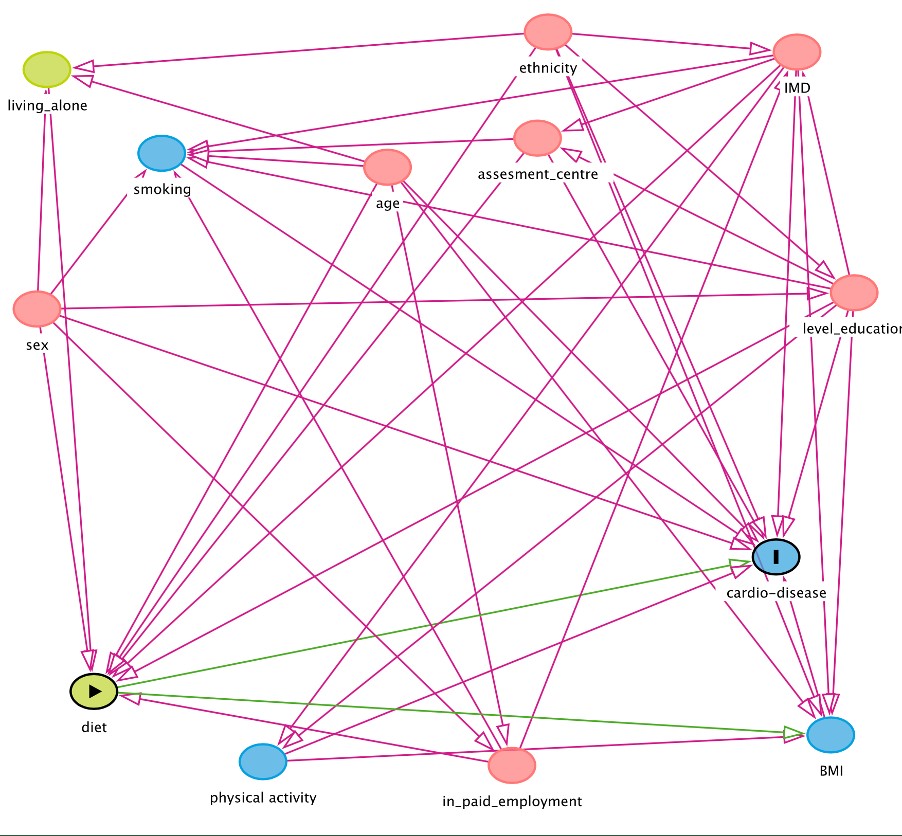

Supplement: Supplementary_material_figure_1_fdad103 [file supplementary_material_figure_1_fdad103.jpeg]
