# Supplementary material for: Development of a measure of dietary quality for the UK Biobank
Source: J Public Health (Oxf). 2023 Jun 29;45(4):e755–62. doi: 10.1093/pubmed/fdad103 (PMC10687865; doi:10.1093/pubmed/fdad103)
Supplement: Supplementary_material_table_1_fdad103 [file supplementary_material_table_1_fdad103.docx]

**Table 1. Flowchart for how to create the FFQ dietary quality score for UK Biobank participants**

These values are based on analyses performed participants of the UK Biobank cohort

| **Food item** | **Mean** | **SD** | **Coefficient** | **Units** |
| --- | --- | --- | --- | --- |
| Cooked vegetable (var=1289) | 20.3 | 15.9 | -0.1509 | tablespoons/day converted into tablespoons/week |
| Salad/raw vegetable (var=1299) | 17.5 | 17.8 | -0.1832 | tablespoons/day converted into tablespoons/week |
| Fresh fruit (var 1309) | 16.2 | 12.4 | -0.1859 | pieces/day converted into pieces/week |
| Dried fruit (var=1319) | 7.10 | 14.3 | -0.1551 | pieces/day converted into pieces/week |
| Oily fish (var=1329) | 1.7 | 0.9 | 0.0232 | N. times per week |
| Non-oily fish (var=1339) | 1.7 | 0.8 | 0.1054 | N. times per week |
| Processed meat (var=1349) | 1.7 | 1.1 | 0.4101 | N. times per week |
| Poultry (var=1359) | 2.2 | 1.0 | 0.3547 | N. times per week |
| Beef (var=1369) | 1.3 | 0.9 | 0.4116 | N. times per week |
| Lamb/mutton (var=1379) | 1.2 | 0.8 | 0.3795 | N. times per week |
| Pork (var=1389) | 1.0 | 0.8 | 0.3903 | N. times per week |
| Cheese (var=1408) | 2.6 | 1.2 | 0.0499 | N. times per week |
| Tea (var=1488) | 21.5 | 18.8 | 0.0402 | cups/day converted into cups/week |
| Coffee (var=1498) | 12.2 | 12.9 | 0.0877 | cups/day converted into cups/week |
| Water (var=1528) | 23.3 | 17.4 | -0.1485 | Glasses/day converted into glasses/week |
| Bread* (var=1438) |  |  |  | Slices/week |
| Cereal± (var=1458) |  |  |  | Bowls/week |
| Brown/wholemeal bread | 8.8 | 8.4 | -0.0785 | Slices/week |
| White bread | 2.6 | 6.3 | 0.1897 | Slices/week |
| High fibre cereal | 4.3 | 2.7 | -0.1360 | Bowls/week |
| Low fibre cereal | 0.7 | 1.9 | 0.1383 | Bowls/week |

* Bread (var=1438) was combined with Bread type (var=1448) to derive slices/week of brown/wholemeal bread or white bread.

± Cereal (var=1458) was combined with Cereal type (var=1468) to derive either high fibre cereal (All Bran, Branflakes, Biscuit cereals, Muesli, Oat) or low fibre cereal (Cornflakes, Frosties)

Steps to derive diet quality score

1. For each variable in a new dataset, ensure the data are in the units described in the table.
2. Check that the means and SDs of each variable are (very) approximately the same as those in the table. If they are not this may indicate a problem with the units.
3. Create the diet quality score by multiplying the coefficient for each food and drink by the individual’s frequency of consumption and then summing this value across all 19 items
4. Multiply the obtained score by 1 so that positive values of the score identify healthy items
5. Transform the score using Fisher-Yates normal scores to obtain a variable with a mean of 0 and a standard deviation of 1.
