# Supplementary material for: Development of a measure of dietary quality for the UK Biobank
Source: J Public Health (Oxf). 2023 Jun 29;45(4):e755–62. doi: 10.1093/pubmed/fdad103 (PMC10687865; doi:10.1093/pubmed/fdad103)
Supplement: Supplementary_material_table_2_fdad103 [file supplementary_material_table_2_fdad103.docx]

| **Table 2.** Principal component analysis coefficients for component 1 | | |
| --- | --- | --- |
| **Food or drink item** | **Component 1 "unhealthy diet score"Ʇ** | |
| Beef | | -0.4116* |
| Processed meat | | -0.4101 |
| Pork | | -0.3903 |
| Lamb | | -0.3795 |
| Poultry | | -0.3547 |
| White bread | | -0.1897 |
| Low fibre cereals | | -0.1383 |
| Non oily fish | | -0.1054 |
| Coffee | | -0.0877 |
| Cheese | | -0.0499 |
| Tea | | -0.0402 |
| Oily fish | | -0.0232 |
| Brown/wholemeal bread | | 0.0785 |
| High fibre cereals | | 0.136 |
| Water | | 0.1485 |
| Cooked vegetables | | 0.1509 |
| Dried fruit | | 0.1551 |
| Salad/raw vegetables | | 0.1832 |
| Fresh fruit | | 0.1859 |
| Percentage of variation explained | | 14% |
