# Supplementary material for: Development of a measure of dietary quality for the UK Biobank
Source: J Public Health (Oxf). 2023 Jun 29;45(4):e755–62. doi: 10.1093/pubmed/fdad103 (PMC10687865; doi:10.1093/pubmed/fdad103)
Supplement: Supplementary_material_table_3_fdad103 [file supplementary_material_table_3_fdad103.docx]

| **Table 3.** Smoking and physical activity compared by dietary quality | | | |
| --- | --- | --- | --- |
|  | Categories of diet score | | |
|  | **unhealthy diet (score<=0)** | **healthy diet**  **(score >0)** | p-value* |
| Current smoker, N (%) | 1,924 (11.6) | 1,271 (7.7) | <0.001 |
| More than 1 days/week vigorous physical activity, N (%) | 7,737 (48.5) | 8,820 (55.2) | <0.001 |
| *p-value estimated with chi-squared test | | | |
